# Role of non-coding-RNAs in response to environmental stressors and consequences on human health

**DOI:** 10.1016/j.redox.2020.101580

**Published:** 2020-07-18

**Authors:** Verónica Miguel, Santiago Lamas, Cristina Espinosa-Diez

**Affiliations:** a Programme of Physiological and Pathological Processes, Centro de Biología Molecular "Severo Ochoa" (CSIC-UAM), Madrid, Spain; b Pittsburgh Heart, Lung, Blood, and Vascular Medicine Institute, University of Pittsburgh, PA, USA

## Abstract

Environmental risk factors, including physicochemical agents, noise and mental stress, have a considerable impact on human health. This environmental exposure may lead to epigenetic reprogramming, including changes in non-coding RNAs (ncRNAs) signatures, which can contribute to the pathophysiology state. Oxidative stress is one of the results of this environmental disturbance by modifying cellular processes such as apoptosis, signal transduction cascades, and DNA repair mechanisms. In this review, we delineate environmental risk factors and their influence on (ncRNAs) in connection to disease. We focus on well-studied miRNAs and analyze the novel roles of long-non-coding-RNAs (lncRNAs). We discuss commonly regulated lncRNAs after exposure to different stressors, such as UV, heavy metals and pesticides among others, and the potential role of these lncRNA as exposure biomarkers, epigenetic regulators and potential therapeutic targets to diminish the deleterious secondary response to environmental agents.

## Highlights

• Environmental stressors induce epigenetic changes that lead to long-lasting gene expression changes and pathology development.
• NcRNAs, miRNAs and lncRNAs, are epigenetic modifiers susceptible to changes in expression after environmental insults .
• LncRNAs influence cell function partnering with other biomolecules such as proteins, DNA, RNA or other ncRNAs.
• LncRNA dysregulation affects cell development, carcinogenesis, vascular disease and neurodegenerative disorders.
• ncRNA signatures can be potentially used as biomarkers to identify exposure to specific environmental stressors.

## Graphical abstract

ncRNA dysregulation after toxic exposure. Different environmental stressors induce epigenetic changes in DNA, such as the decrease in DNA methylation, the activation of transcriptional mechanisms like the Nrf2 pathway or the alteration of ncRNAs (microRNAs or LncRNAs) expression as an interactive network. Cellular dysregulation of ncRNAs can accelerate mechanisms of disease and malignancy like cancer, vascular disease or neurogenerative disease. Created with Biorender.com.

**Figure:**
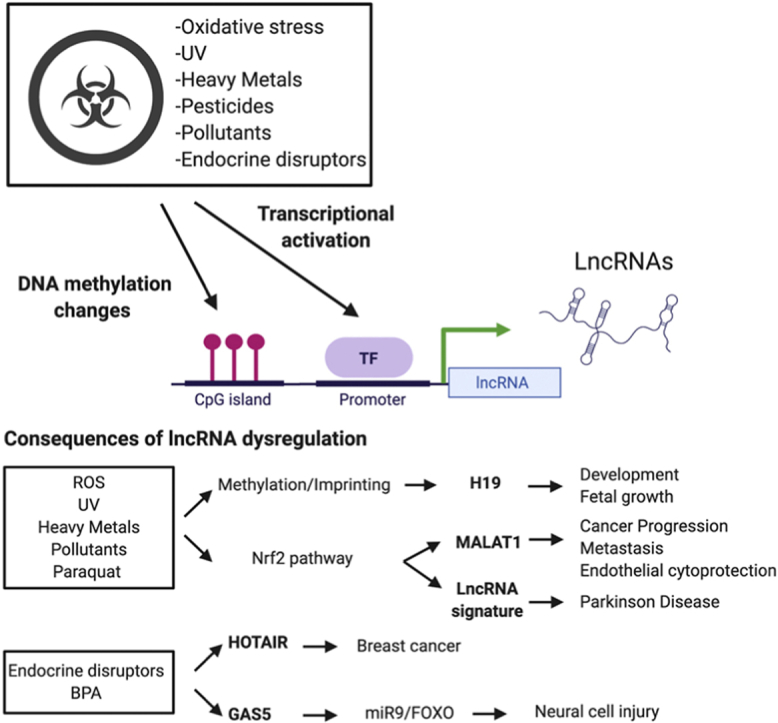

## List of abbreviations in alphabetical order

AIRN: antisense Igfr2 non-coding RNA
AML: acute myeloid leukemia
As: arsenic
ATF4: activating transcription factor 4
ATO: Arsenic trioxide
BARD: BRCA1 associated RING domain 1
BNIP3: BCL2 interacting protein 3
BPA: Benzene and bisphenol A
BPF: bisphenols F
BPS: bisphenols S
CAR: constitutive active-androstane receptor
Cd: cadmium
CD1D: cluster of differentiation 1
CDKN1A: Cyclin-dependent kinase inhibitor 1
CeRNA: competing endogenous RNA
CFTR: cystic fibrosis transmembrane regulator
CNS: central nervous system
COPD: chronic obstructive pulmonary disease
CSE: cigarette smoke extract
DACT3.AS1: dishevelled binding antagonist of beta catenin 3 antisense RNA 1
DAPK1: death-associated protein kinase 1
DDB1: damage specific DNA binding protein 1
DDB2: damage specific DNA binding protein 2
DEET: N,N-diethyl-m-toluamide
DES: diethylstilbestrol
DGCR8: DGCR8 microprocessor complex subunit
DICER: double-stranded RNA (dsRNA) endoribonuclease
DLX6: distal-less homeobox 6
DNMT3b: DNA methyltransferase 3 beta
DPP10: dipeptidyl peptidase like 10
DROSHA: double-stranded RNA-specific endoribonuclease
EBs: embryoid bodies
EMT: epithelial to mesenchymal transition
ERCC1: ERCC excision repair 1
EZH2: enhancer of zeste 2 polycomb repressive complex 2 subunit
GAS5: growth arrest specific 5
GDM: gestational diabetes mellitus
GEMIN4: gem nuclear organelle associated protein 4
GKN2: gastrokine 2
GR: glucocorticoid receptor
HBE: human bronchial epithelial
HIF-2α: hypoxia-inducible factor 2-alpha
HNF1A-AS1: hepatocyte nuclear factor 1A antisense-RNA-1
HNF4A-AS1: hepatocyte nuclear factor 4A antisense-RNA-1
HOTAIR: hox antisense intergenic chromatin markers RNA
HOTAIRM1: HOXA Transcript Antisense RNA Myeloid-Specific 1
HOTTIP: HOXA distal transcript antisense RNA
HOX: homeobox
IGF2: insulin like growth factor 2
IGFR2: insulin like growth factor 2 receptor
JNK: c-Jun N-terminal kinase
KCNQ1: potassium voltage-gated channel subfamily Q member 1
Keap1: Kelch-like ECH-associated protein 1
LCPAT1: lung cancer progression-association transcript 1
lncRNA: long-non-coding-RNA
MALAT1: Metastasis Associated Lung Adenocarcinoma Transcript 1
MAPK: MKK3/p38/mitogen-activated protein kinase
MBD1: methyl-CpG–binding domain protein 1
MEG3: maternal expression gene 3
miRNAs: microRNAs
MSH2: mutS homolog 2
NAC: N-acetylcysteine
ncRNAs: non-coding RNAs
NORAD: Non-coding RNA activated by DNA damage
Nrf2: nuclear factor erythroid 2–related factor 2
NT: nucleotide
OGG1: 8-oxoguanine DNA glycosylase
ORF: Open reading frame
OSGIN1: oxidative stress-induced growth inhibitor 1
PANDAR: promoter of CDKN1A antisense DNA damage activated RNA
Pb: lead
PBDEs: polybrominated diphenyl ethers
PBLs: peripheral blood leukocytes
PCAWG: Pan-Cancer Analysis of Whole Genomes
PCBs: polychlorinated biphenyls
PEG3: paternal expression gene 3
PFCAs: perfluoro carboxylic acids
PFOS: perfluorooctanesulfonate
piRNAs: piwi-interacting RNAs
PKM2: pyruvate kinase muscle isoform 2
PM: particulate matter
Pol II: RNA polymerase II
PPARA: peroxisome proliferator activated receptor alpha
pre-miRNA: precursor miRNA
PXR: pregnane X receptor
RAD50: RAD50 Double Strand Break Repair Protein
RedoximiRs: redox-responsive microRNAs
RISC: RNA-induced silencing complex
ROS: reactive oxygen species
RPL37: ribosomal protein L37
siRNAs: short interfering RNAs
Skp2: S-phase kinase protein 2
SRBP1: sterol regulatory element-binding protein
TGF-β: transforming growth factor-beta
UBA52: ubiquitin A-52 residue ribosomal protein fusion Product 1
UCA1: urothelial cancer associated 1
UTR: untranslated region
UV: ultraviolet
VDAC1: voltage-dependent anion channel 1
VEGF: vascular endothelial growth factor
Xist: X-inactive specific transcript
XRCC1: X-ray repair cross complementing 1
Zeb1: Zinc finger E-box-binding homeobox 1
Zeb2: zinc finger E-Box binding homeobox 2

## 1 Introduction

Mammalian genes are susceptible to changes depending on their environment, influencing disease development. These environmental stressors promote perdurable epigenomic changes. Non-coding RNAs are epigenetic modifiers of gene expression that have been in the spotlight for the past decades. Initially misconceived as junk DNA, these RNAs do not translate into functional proteins. The discovery and characterization of ncRNAs has shed light on the complexity and depth of gene regulation [1]. Depending on their length and function, they are classified as small non-coding RNAs and long-non-coding RNAs. Those shorter than 200 nucleotides (nt) are considered short non-coding RNAs and include microRNAs (miRNAs), short interfering RNAs (siRNAs) and piwi-interacting RNAs (piRNAs), whereas those longer than 200 nt are termed long non-coding RNAs (lncRNAs) [2].

While the function of piRNAs and siRNAs is related to maintaining genomic stability in the germline and anti-viral responses respectively, multiple microRNAs and lncRNAs have been involved in the development and progression of different diseases [3,4]. LncRNAs can act as positive or negative regulators of gene expression through transcriptional and post-translational regulatory mechanisms. They may target processes such as chromatin structure, RNA maturation and protein synthesis and transport [5,6]. This review summarizes our current understanding of the role of microRNAs and lncRNA in pathophysiological changes caused by exposure to environmental stressors.

## 2 Non-coding-RNA biogenesis and function

### 2.1 MicroRNAs

miRNAs are a large family of conserved, small, non-coding RNAs of ~22 nucleotide (nt) length. The human miRNAome is composed of 1,917 precursor microRNAs (pre-miRNAs) and 2,654 mature miRNAs, which regulate at least 60% of protein-coding genes by repressing the translation and/or inducing degradation of their messenger RNA (mRNA) targets [7]. Their mechanism of action is based on the Watson-Crick base-pair complementarity between miRNAs and target sequences mainly located in the 3′ untranslated region (UTR) of mRNAs [8]. MicroRNAs expression patterns differ among tissues and cell types. miRNA-mRNA interaction depends on several factors such as subcellular location, abundance of the miRNAs and/or their target mRNAs, and the affinity between the miRNA-mRNA sequences [9].

miRNAs are transcribed by RNA polymerase II (Pol II) as a transcript precursor RNA known as primary miRNA (pri-miRNA). This pri-miRNA is sequentially processed by an RNAse III double-stranded RNA-specific endoribonuclease (DROSHA) in the nucleus, resulting in a ~70 nt pre-miRNA. The pre-miRNA is exported to cytoplasm through Exportin-5 where a second RNAse III, the Double-stranded RNA endoribonuclease (DICER), generates the ~22-nt mature miRNA. miRNAs associate with specific mRNAs within the RNA-induced silencing complex (RISC), providing sequence-specific silencing activity [10]. One strand of the mature miRNA (the ‘guide’ strand) is loaded into RISC whereas the other strand is discarded [8]. A single miRNA can potentially target hundreds or thousands of mRNAs, regulating crucial functions in numerous biological processes including development, differentiation, stress response and apoptosis [11].

### 2.2 LncRNAs

LncRNAs are untranslated transcripts longer than 200 nts [12]. Human lncRNAs spans are estimated to be around 127,802 transcripts grouped on 56,946 lncRNA genes in the last LNCipedia release [13]. However, some evidences indicate that only 1,000 lncRNAs would be present at greater than one copy per cell [14]. LncRNAs' mechanisms of action include *1) promoter-specific repression or activation of transcription, 2) modulation of the epigenetic profile, 3) regulation of mRNA stability or 4) miRNA function by acting as sponges* [5]. LncRNA expression is tissue-specific, and they have a tight regulation during development. They can have a stand-alone promoter, and they are usually affected by DNA methylation changes. Although the primary sequence is poorly conserved, lncRNA secondary and tertiary structure has been well conserved across mammals. The first case of lncRNA regulation was described in the 1990s. The lncRNA H19 was described as an "unusual RNA molecule" [13], and the lncRNA X-inactive specific transcript (Xist) was characterized as a gene without a conserved open reading frame (ORF), suggesting that it may function as a scaffold RNA in the nucleus [15,16]. These were the first hints indicating that not only the sequence, but the RNA structure and folding would have gene regulation roles.

#### 2.2.1 LncRNA classification

The diversity of lncRNAs, in terms of their location, structure, and function, makes their classification challenging. They can be classified into five groups regarding genomic location: *1) stand-alone lncRNAs, 2) natural antisense transcripts, 3) pseudogenes, 4) long-intronic RNAs, and 5) promoter-associated RNAs or enhancers* [17]. *Stand-alone lncRNAs* do not overlap with protein-coding genes and they are usually referred as long-intergenic ncRNA (lincRNAs). These lncRNAs are transcribed by Pol II, polyadenylated and have multiple splicing isoforms. *Natural antisense transcripts* are transcribed in the opposite strand of a coding-gene. Interestingly, this happens in several imprinted regions like insulin like growth factor 2 receptor (Igf2r) and potassium voltage-gated channel subfamily Q member 1 (Kcnq1). *Transcribed pseudogenes* have now been shown to act as gene expression regulators. *Long-intronic ncRNAs* are encoded through the introns of annotated genes, however, their study in mammals awaits further characterization. Finally, *promoter associated RNAs* are a heterogenous group of ncRNA transcribed from transcription start sites in both, sense and antisense, directions [17].

Considering their regulatory function, lncRNAs can be classified into those that act in cis, regulating nearby genes, or those that regulate different biological functions in trans, in distant regions from their genomic location [18].*Cis-acting lncRNAs* regulate the expression of the proximal genes. The best example of this type of cis-acting lncRNA is the X-inactive specific transcript (Xist), which silences one of the X chromosomes in females for dosage compensation, due to the presence of repetitive domains. In other cases, the function of the lncRNA is independent of its transcription [19,20]. A clear case is the antisense Igfr2 non-coding RNA (AIRN) that generates transcriptional interference with the imprinted Igfr2 gene, silencing the paternal allele [21]. A third case is that the cis-regulation depends on DNA elements within the lncRNA locus. The lincRNA-p21 contains DNA elements that regulate the neighbor gene Cyclin-dependent kinase inhibitor 1 (CDKN1A) in a p53 dependent manner [22,23].*Trans-acting lncRNAs* leave the site of transcription to influence chromatin organization, like the HOX (homeobox) antisense intergenic chromatin markers RNA (HOTAIR) that is required to maintain repressive chromatin marks at the distant HOXD locus [24]. Likewise, lncRNA can interact with protein or other RNA molecules. They can serve as a decoy for RNA-binding proteins like the case of the lncRNA NORAD (Non-coding RNA activated by DNA damage) [25,26]. They can also regulate the abundance or other lncRNAs molecules like their counterpart microRNAs; these lncRNAs are known as competing endogenous RNAs (ceRNAs) [27].

## 3 Roles of ncRNAs in environmental health

Exposure to environmental agents is long-lasting and triggers epigenetic changes. Abnormal expression of ncRNAs can induce developmental changes or lead to disease progression. We discuss the most common environmental hazards and their involvement in miRNA and lncRNA biology along with the role of these aberrantly expressed ncRNAs in different related pathologies (Graphical abstract).

### 3.1 Oxidative stress

In previous works, we summarized how redox-responsive microRNAs (redoximiRs) react to environmental stressors and their involvement in cardiovascular disease and fibrosis development [[28], [29], [30]]. The nuclear factor erythroid 2–related factor 2 (Nrf2) pathway is a key modulator of these redoximiRs [31]. Similarly, numerous lncRNAs have been associated with dysregulation of the Nrf2/Keap1 pathway. Thus, the lncRNA Metastasis Associated Lung Adenocarcinoma Transcript 1 (MALAT1) is induced by hydrogen peroxide in endothelial cells and acts as a cytoprotector lowering Keap1 (Kelch-like ECH-associated protein 1) levels and stabilizing the antioxidant transcription factor Nrf2 through direct interaction with these factors [32]. The expression of MALAT1 is regulated by perturbation of redox homeostasis, generated by hypoxic or ischemic conditions as well [33,34]. MALAT1 also dampens endogenous microRNAs and promotes changes in the redox state. Moreover, MALAT1 regulates apoptosis in mesenchymal cells after treatment with the antioxidant quercetin [35]. The lncRNA H19 is also sensitive to hydrogen peroxide. In cardiac progenitor cells exposed to hydrogen peroxide, H19 is decreased. Interestingly, miR-365, a microRNA derived from H19, is reduced [36]. H19 overexpression reduces oxidative stress in a diabetic mouse model by targeting miR-657 to inhibit voltage-dependent anion channel 1 (VDAC1). This mechanism has shown benefit in cancer cells, as inhibition of H19 increases oxidative stress on hepatocellular carcinoma and counteracts chemotherapy resistance [37]. A similar effect was found on hippocampal neurons in diabetic mice, through inhibition of H19 that led to decreased insulin like growth factor 2 (IGF2) gene methylation [38].

Metabolic activation and detoxification reactions catalyzed by cytochrome P450 enzymes (CYPs) affect the toxicities of many xenobiotic compounds. CYP-mediated toxicity is associated with two types of reactions that either produce reactive electrophiles that damage critical biomolecules or induce oxidative stress via free radical reactions. ncRNAs are involved in regulating CYP expression. For instance, CYP450 family 2 subfamily E member 1 (CYP2E1) mRNA is directly targeted by multiple miRNAs at different regions. miR-214–3p targets CYP2E1 at the coding sequence, while miR-552, miR-570, and miR-378a-5p target its 3′-UTR [39]. The lncRNAs hepatocyte nuclear factors 1A antisense-RNA-1 (HNF1A-AS1) and 4A antisense-RNA-1 (HNF4A-AS1) are important regulators of CYP gene expression as well, interfering with the xenobiotic-dependent CYP2E1 bioactivation [40].

### 3.2 Ultraviolet radiation

Exposure to Ultraviolet (UV) radiation is one of the leading causes of skin disease development like skin cancer, skin aging, eye damage and immune system suppression. While some of these effects are related with an increased oxidative stress due to UV radiation, some lncRNAs have been connected only to this specific environmental stressor. Yo et al., demonstrated that due to the differential cellular effects of UVA versus UVB, lncRNA signatures from UVA and UVB exposed keratinocytes only partially overlapped [41] . Kraemer et al. analyzed the miRNA UVA and UVB signature in human keratinocytes as well. They defined a 10-miRNA overlapping set that includes miR-330–3p, miR-988, miR-598, miR-376, miR-323–3p, miR-494, miR-376c, miR-191, miR-501–5p and miR-96. In this study, the authors described a significant upregulation of miR-23b, a miRNA responsible for keratinocyte differentiation in human skin. These results suggest that UVA through miR-23b induces an accelerated keratinocyte differentiation [42].

Some lncRNAs are upregulated in response to UV to rein in the damage response. This is the case of the lncRNA RP11‐670E13.6 in dermal fibroblasts. Inhibition of this specific lncRNA leads to senescence through a p16/pRB-dependent mechanism. Therefore, increased expression of this lncRNA potentially delays senescence due to DNA damage in fibroblasts exposed to UV radiation [43]. LncRNAs dysregulated by UVB such as lnc-GKN2-1:1 and lnc-CD1D-2:1 showed a potential role in cancer progression. Lnc-CD1D-2:1 are induced after UVB irradiation and the use of the broad antioxidant N-acetylcysteine (NAC) can impair the reactive oxygen species (ROS) dependent upregulation in melanocytes [44]. In dermal fibroblasts, MALAT1 was upregulated after UVB. However, in keratinocytes its expression was not affected, highlighting the cell specificity of these mechanisms [45].

### 3.3 Persistent environmental chemicals

Involvement of ncRNAs in xenobiotic-induced disease pathogenesis has been mostly covered for persistent environmental chemicals including polychlorinated biphenyls (PCBs), polybrominated diphenyl ethers (PBDEs), perfluoro carboxylic acids (PFCAs), perfluorooctanesulfonate (PFOS), benzene and bisphenol A (BPA). Summary of ncRNA studies on persitent environmental chemical can be found on Table 3.

**Table 1:** List of relevant studies in heavy metals and microRNAs from 2016 to present.

	Target genes	Altered cell function	Species or cell type	References
**miR-31 ↓**	Special AT-rich sequence-binding protein 2 (SATB2)	Cell growth, migration, invasión	Human bronchial epithelial (BEAS-2B) cells	[84]
**miR-330 ↑**	S-phase kinase associated protein 2 (Skp2)	Cell growth, motility, invasión, apoptosis	Pancreatic cancer (PC) cells	[85]
**miR-122 ↑**	Pyruvate kinase M2	Autophagy	Primary hepatocytes	[86]
**miR-4665 ↑**	Gse1 Coiled-Coil Protein (GSE1)	Migration, invation, EMT	Gastric cancer cell lines	[87]
**miR-155 ↑**	Nrf2/NQO1/HO-1 Bcl-2/Bax	Cell survival, migration, apoptosis	Human lung adenocarcinoma A549R cell line	[88]
**miR-539 ↓**	Bcl-2/Bax	Apoptosis	Hepatocellular carcinoma	[89]
**miR-372 ↓**	Large tumour suppressor kinase 2 (LATS2)	Cell proliferation and migration	Human prostate cancer cell lines DU145 and PC3	[90]
**miR-214 ↓**	Activating Transcription Factor (4ATF4) and Enhancer Of Zeste Homolog 2 (EZH2)	Cell survival	Mouse erythroleukemia (MEL) cells	[91]
Cadmium				
**miR-125 ↓**	Bak and caspase-3	Apoptosis	Renal epithelial cell line LLC-PK1	[92]
**miR-33 ↓**	AMP-activated protein kinase (AMPK)	Autophagy	Chicken spleen	[93]
**miR-30 ↓**	Glucose regulated protein (GRP78)	Autophagy	Chiken kidney	[94]
**miR-181 ↓**	Toll-like receptor 4 (TRL4) and sequestosome 1 (SQSTM1)	inflammation	Human bronchial epithelial (BEAS-2B) cells	[95]
**miR-122 ↑**	Phospholipase D1 (PLD1)	Apoptosis	Human renal cell lines HK-2 and NRK-52E	[96
**miR-326 ↑**				
Mercury				
**miR-92 ↑**	N/A	N/A	Human plasma	[97]
**miR-486 ↑**				
**miR-575 ↓**	N/A	N/A	Human cervical cells	[98]
**miR-4286 ↓**

**Table 2:** List of relevant studies in heavy metals and lncRNAs from 2015 to present.

	Target genes	Altered function	Cell/Tissue	Ref
Arsenic				
**UCA1 ↑**	Oxidative stress induced growth inhibitor 1 (OSGIN1) miR-184	Autophagy, cell death	Hepatocellular carcinoma	[99]
**Kcnq1ot1 ↓**	Kcnq1	Electrophysiology	Cardiac muscle	[100]
**MEG3 ↑**	Pyruvate kinase muscle isoform 2 (PKM2)	EMT	Hepatocellular carcinoma cell lines	[101]
**PU.1 AS ↑**	Enhancer of Zeste Homolog 2 (EZH2)	Lipid homeostasis	Hepatocyte cell line NCTC	[102]
**PANDAR ↑**	N/A	DNA damage	Peripheral blood lymphocytes	[103]
		DNA methylation		
**MALAT1 ↑**	Hypoxia-inducible factor-2 α (HIF-2α)	Cell migration inflammation	Hepatocellular carcinoma	[104]
Cadmium				
**ENST00000414355 ↑**	ATM, ATR, ATRIP DDB1, DDB2, OGG1, ERCC1, MSH2, RAD50, XRCC1, BARD1	DNA damage and repair	Lung tissue and bronchial epithelial cells (16HBE)	[105]
**MALAT1 ↑**	FOXC2, STAT, BAX, EGFR, TGF-β, BCL-2	Cell proliferation, apoptosis, migration and invasion	Lung tissue and bronchial epithelial cells (16HBE)	[106]
**MT1DP ↑**	NRF2, miR-365	Oxidative stress, cell death	Hepatocellular carcinoma cell line HepG2	[107, 108]
	RhoC, MT1H			
Mercury				
**MALAT ↑**	N/A	Neurotoxicity	Brain	[109]
**H19 ↓**	N/A	Development	Sperm	[110]

**N/A**: not available.

**Table 3:** List of relevant studies in persistent environmental chemicals and ncRNAs from 2013 to present.

	Target genes	Altered function	Cell/Tissue	Ref
PBCs and PBDEs				
**miR-191↑**	N/A	Fetal malformations	Human PBMCs	[128]
**miR-132↑**	p250GAP	Neuropsychological dysfunctions	Hippocampal neurons	[129]
**miR-145↑**	N/A	Oxidative stress, development, apoptosis	Human embryonic stem cells	[[50], [51]]
**miR-335↑**				
PFCAs and PFOs				
**miR-34↑**	Ldha, Fut8	Hepatotoxicity	Mouse liver	[[54], [55]]
**miR-362-3p↓**				
**miR-338-3p↓**				
**miR-155↑**	Nrf2	Adipogenesis, Antioxidant pathways	SD Rat and HepG2	[[130], [131]]
Benzene and Bisphenol				
**miR-21↑**	p38/MAPK	Obesity	Preadipocyte	[66],[67]
**miR-19↑**	PTEN/AKT/p53			
**HOTAIRM1↓**	N/A	AML	Human lymphoblast	[58]
**GAS5↓**	Jun/RAS	Neurotoxicity	PC12 cells	[75]

**PBMCs**: peripheral blood mononuclear cells.

**SD**: Sprague Dawley.

**AML**: acute myeloid leukemia.

**N/A**: not available.

#### 3.3.1 Polychlorinated biphenyls and polybrominated diphenyl ethers

PCBs are associated with several vascular alterations such as endothelial cell dysfunction and atherosclerosis, by producing oxidative stress and induction of proinflammatory cytokines and cell adhesion proteins [46]. In human peripheral blood mononuclear cells, miR-191 expression correlates with total blood concentrations of the dioxin-like congener PCB169 [47]. The nondioxin-like PCBs have been associated with neuropsychological dysfunctions such as Rett syndrome and schizophrenia through the modulation of miR-132 [48]. In primary human endothelial cells, the commercial PCB mixture Aroclor 1260 modifies the expression of 21 miRNAs associated with vascular diseases [49]. The polybrominated diphenyl ether BDE-209 reduces pluripotent gene expression via epigenetic regulation, including the modulation of miR-145 and miR-335 and the triggering of apoptosis through ROS generation [50,51]. Interestingly, the liver needs the gut microbiota to efficiently biotransform chemicals such as PBDE. Yanfei Li et al., described the changes in lncRNA signatures between germ free (sterile) and conventional mouse. They described how lack of the gut microbiota increased the number of PBDE modulated lncRNA in mouse liver and these lncRNA signatures were able to discriminate between different PBDE species such as BDE-47 and BDE-99 [52]. Similarly, Zhang et al., analyzed the expression profile of lncRNA in the human liver cell line HepaRG. They described how PBDE exposure affect lncRNA expression and lncRNA-protein interactions in a (PXR)/(CAR) dependent and independent manner [53].

#### 3.3.2 Perfluoro carboxylic acids and perfluorooctanesulfonate

The perfluorocarboxylic acid PFNA generates hepatomegaly, increases hepatic triglycerides and total cholesterol, and enhances serum transaminases at least partially through the modulation of miR-34a, miR-362–3p, and miR-338–3p [54,55]. PFOS induces adipogenesis and glucose uptake in preadipocytes. These changes correlates with miR-155-dependent activation of the oxidative stress-responsive transcription factor Nrf2, which is essential for upregulating antioxidant genes and metabolic reprogramming [54,56].

#### 3.3.3 Endocrine disruptors, benzene and bisphenol

Benzene exposure positively correlates with hematological disorders, affecting the expression of microRNAs that control pathways involved in cell proliferation and differentiation, including vascular endothelial growth factor (VEGF), transforming growth factor-beta (TGF-β) and Wnt signaling. In peripheral blood mononuclear cells from benzene-exposed workers, six upregulated miRNAs (miR-34a, miR-205, miR-10b, let-7d, miR-185 and miR-423-5p-2) and seven downregulated miRNAs (miR-133a, miR-543, hsa-miR-130a, miR-27b, miR-223, miR-142–5p and miR-320b) were observed, with identified targets involved in these signaling pathways. It also highlights their potential role as biomarkers of chronic benzene poisoning [57]. The lncRNA HOXA Transcript Antisense RNA, Myeloid-Specific 1 (HOTAIRM1) has shown a leading role in the development of acute myeloid leukemia (AML). In long-term benzene-exposed workers HOTAIRM1 expression is decreased. Benzene exposure triggers DNA Methyltransferase 3 Beta (DNMT3b) expression and hypermethylation of the HOTAIRM1 promoter region [58].

Bisphenol A (BPA) is an endocrine disruptor that can act as an agonist or antagonist through estrogen receptor signaling. BPA exposure is involved in the development of hormone-dependent cancers in breast, prostate, and ovarian [59]. Several studies have shown that exposure to BPA can lead to epigenetic modifications in the placenta that may have an impact on the fetus [60]. Although the studies are limited, BPA affects gene imprinting in the placenta and global and CpG methylation [61]. miR-146 expression has been proposed as a biomarker for developmental exposure to BPA [62]. miR-134 mediates BPA-mediated disturbances in pluripotency in embryonic stem cells and embryoid bodies [63]. Furthermore, exposure to BPA is associated with breast cancer. In the ER-positive and hormone-sensitive human MCF-7 breast cancer cell line, BPA promotes ER transcriptional activity, which alters the expression profiles of certain miRNAs, including miR-21, an onco-miR frequently upregulated in solid tumors [64]. In addition, BPA induces the expression of miR-19a/b and downregulates the miR-19/PTEN/AKT/p53 axis [65]. BPA exposure has been correlated with type-2 diabetes, gestational diabetes mellitus (GDM) and obesity [66]. miR-21a-5p promoted BPA-induced obesity *in vivo* while BPA-induced preadipocyte differentiation is repressed by miR-21a-5p by targeting map2k3 in the MKK3/p38/mitogen-activated protein kinase (MAPK) pathway [67].

Among persistent environmental chemicals affecting lncRNAs, BPA exposure has the strongest impact [68,69]. Thus, the imprinted cluster H19/IGF2 methylation is altered in the placenta. IGF2 gene is located upstream of H19 and is paternally expressed. H19 is a lncRNA expressed from the maternal strand and hypermethylation of this ncRNA has been previously connected to changes in fetal growth [70]. H19 itself controls several genes within the imprinted gene network, recruiting methyl-CpG–binding domain protein 1 (MBD1) as a partner. H19/MBD1 partnership is required for a fine tune regulation of gene expression in the embryo [71]. Therefore, changes in the levels of H19 due to BPA can trigger severe complications in development.

Endocrine disruptors can also affect other lncRNAS such as HOTAIR. HOTAIR has been previously associated with different types of cancer, such as lung, hepatocellular carcinoma, prostate, gastric, and breast cancer [72]). In breast cancer, BPA and diethylstilbestrol (DES) exposure can induce HOTAIR expression *in vitro* and *in vivo*, revealing a new epigenetic mechanism for endocrine disruption in breast cancer [73]. BPA also has adverse effects on the nervous system during neurological development, affecting behavioral problems [74]. Pang et al., described that BPA increases apoptosis on PC12 cells at concentrations higher than 40 μM. Using microarray expression profiling, they characterized 151 differentially expressed lncRNAs in response to BPA. They identified growth arrest specific 5 (GAS5), that controls the response to neural cell injury through regulation of the Jun/RAS axis and is downregulated after BPA treatment [75].

BPA analogues, bisphenols F (BPF), and S (BPS) can promote endocrine disruption properties in specific contexts [76]. Low doses of BPF and BPS, similarly to BPA exposure compromise ncRNA expression in adipocytes *in vitro* [77], so further investigation is needed to understand the toxicity of these chemicals and their relevance in human health. Other endocrine-disrupting chemicals, phthalates, can cross the placenta and affect the fetal epigenome, including lncRNA expression. Li D et al., found a strong correlation between the lncRNAs AIRN, DACT3.AS1, DLX6, DPP10, HOTTIP, LOC143666, and LOC91450 in human placenta and a number of maternal urinary phthalate metabolites [78]. Indeed, in an independent study, the Michigan Mother-Infant Pairs (MMIP) cohort, a correlation between infant cord blood DNA methylation changes and maternal levels of endocrine disrupting chemicals phthalate and BPA was found. The study revealed decreases in methylation of IGF2, and peroxisome proliferator activated receptor alpha (PPARA) with increasing phthalate concentrations, which links early exposures with disease risk later in life [79].

### 3.4 Heavy metals

#### 3.4.1 Arsenic

Chronic exposure to arsenic-contaminated waters is a worldwide problem. Arsenic (As) poisoning is associated with several adverse outcomes in human health, such as skin lesions, a broad spectrum of cancers, and other outcomes like cardiovascular disease or diabetes. Arsenic exposure modulates epigenetic changes in DNA methylation and histone acetylation, including changes in microRNAs and lncRNAs [80]. Kong et al. assessed the relationship among microalbuminuria, metals detected in the urineHg, lead (Pb), As, and cadmium (Cd)) and the levels of miR-21, miR-126, miR-155, and miR-221. They found miR-21 and miR-221 were negatively associated with arsenic and lead levels and miR-21 was associated with microalbuminuria. Thus, miR-21 and miR-221 were proposed as biomarkers of kidney function in the context of heavy metal exposure [81].

In a study of pregnant mothers and their infants exposed to arsenic, Rager et al. examined the potential association between the presence of arsenic in drinking water and maternal urine and miRNAs expression in cord blood. There were significant correlations between a number of cord blood miRNAs (let-7a, miR-107, miR-126, miR-16, miR-17, miR-195, miR-20a, miR-20b, miR-454, miR-96, and miR-98) and urinary arsenic. These miRNAs have been linked to cancer and diabetes. Furthermore, there was a decrease in the expression of several immune response-related mRNAs that was attributed to changes in the levels of these miRNAs [82].

Bollati et al. studied the expression of miR-21, miR-146a and miR-222 in peripheral blood leukocytes (PBLs) from steelworkers occupationally exposed to ambient pollution containing arsenic, iron, nickel, lead, cadmium, chromium, and manganese. Both miR-21 and miR-222 were increased after 3 days of exposure, while miR-222 levels were correlated with lead exposure. Conversely, miR-146a was inversely correlated with lead and cadmium exposure. Furthermore, miR-21 increased expression was associated with levels of the oxidative stress marker 8-hydroxyguanine [83]. In Table 1, we have summarized microRNAs involved with arsenic exposure, together with other heavy metals from studies after 2016 (see Table 2).

The role of miRNAs in cancer-related processes such cell survival, motility, invasion, proliferation and apoptosis has gained increasing attention. The oncogenic role of microRNA induction or repression varies in cancer type specific manner. The miR-122/PKM2 autophagy axis protects hepatocytes from arsenite stress via the PI3K/Akt/mTOR pathway [132]. Further, arsenic inhibits prostate cancer cell proliferation and migration through the regulation of large tumor suppressor kinase 2 by repressing microRNA-372 [133], while it promotes the miR-330-mediated S-phase kinase protein 2 (Skp2) associated inhibition in pancreatic cancer cells, favoring these malignant features [134]. Some of them control the oxidative stress underlying these cellular processes. Thus, miR-155 mediates arsenic trioxide resistance by activating Nrf2 and suppressing apoptosis in lung cancer cells [135], while miR-214 protects erythroid cells against oxidative stress by targeting the ATF4 and the enhancer of EZH2 [136].

LncRNA Urothelial Cancer Associated 1 (UCA1) has an essential protective role in arsenic-induced autophagy through its downstream target oxidative stress-induced growth inhibitor 1 (OSGIN1) and acts as a competing ceRNA for miR-184 [137]. Prenatal arsenic exposure modifies lncRNA GAS5 expression in mouse, reprogramming glucocorticoid receptor (GR) downstream gene transcription. Interestingly, Caldwell et al. observed that GAS5 dysregulation was sex-dependent, inasmuch as GAS5 was reduced in male but not in female mice [138]. MALAT1 is also involved in the response to carcinogenic agents like arsenic. Arsenic increases the expression of MALAT1 in human hepatic epithelial cells *in vitro* through a hypoxia-inducible factor 2-alpha (HIF-2α) positive feedback loop accelerating malignant transformation [139]. Arsenic-induced DNA damage increases the expression of the p53-dependent promoter of CDKN1A antisense DNA damage activated RNA (PANDAR), a regulator of cell senescence. He et al. described how DNA methylation in the PANDAR promoter region is affected in arsenic smelting plant laborers [140]. However, the downstream effects of PANDAR in response to arsenic needs further investigation. Likewise, arsenic poisoning induces a disequilibrium in lipid metabolism. Dong et al. demonstrate that PU.1 AS, an arsenic-induced lncRNA, regulates the EZH2/Sirtuin-6 axis by decreasing sterol regulatory element-binding protein (SRBP1) and triglyceride synthesis in the liver [141].

Some arsenic compounds like Arsenic trioxide (ATO) are used in the clinic as chemotherapeutic agents [142]. ATO has shown excellent results in hepatocellular carcinoma (HCC). Fan et al. reported that ATO positively regulates maternal expression gene 3 (MEG3) lncRNA. In this same work, they described that ATO reduces the expression of pyruvate kinase muscle isoform 2 (PKM2), a glycolytic enzyme with a high affinity for arsenic. Through this double regulation, they proposed that MEG3 can control epithelial to mesenchymal transition (EMT), and therefore invasion and metastasis [143]. Some studies have revealed that the treatment of promyelocytic leukemia with ATO triggers secondary cardiovascular effects such as sudden cardiac death. Jiang et al. demonstrated that the lncRNA Kcnq1ot1 is responsible for the ATO-induced long QT syndrome that results in cardiac death [144].

#### 3.4.2 Cadmium

Cd is a highly toxic and persistent heavy metal. Fabbri et al. studied the effects of Cd exposure in the human hepatoma cell line HepG2. They found that higher Cd exposure induced transcriptional changes related with cancer and depressed hepatic function and that some let-7 miRNA family members were differentially expressed after Cd exposure, suggesting a connection between their tumor suppressor roles and cadmium carcinogenesis [145]. Chronic exposure to Cd induces significant alterations of miRNAs in kidneys. Cd-induced apoptosis is modulated by miR-125a/b via the mitochondrial pathway in renal epithelial cells [146]. MiR-122–5p and miR-326–3p may serve as novel biomarkers for Cd-induced nephrotoxicity [147]. Cadmium modulates autophagy through microRNA regulation. Thus, its exposure induces BNIP3-dependent autophagy in chicken spleen by modulating miR-33-AMPK axis [148], while cadmium-mediated miR-30a-GRP78 leads to JNK-dependent autophagy in chicken kidney [149].

Cd is a significant component of cigarette smoke. Hassan et al. showed that cigarette smoke and cadmium exposure increased miR-101 and miR-144 expression in human airway epithelial cells, which suppressed the expression of the cystic fibrosis transmembrane regulator (CFTR) protein. Furthermore, they showed that cigarette smoke exposure induced miR-101 in mice lungs. Moreover, chronic obstructive pulmonary disease (COPD) patients had higher pulmonary expression of miR-101, suggesting a link between cigarette smoking, Cd exposure, and suppression of CFTR in COPD [150].

Chronic Cd exposure also triggers changes in gene and lncRNA expression. Gu et al. recently reviewed the latest findings in lncRNAs related to Cd poisoning [151]). In this context, the role of MT1DP in Cd-induced liver toxicity through inhibition of Nrf2 and RhoC pathway should be emphasized [152,153]. The lncRNA MALAT1 can be used as a potential biomarker for Cd toxicity in the lung. Huang et al. have observed a positive correlation between MALAT1 levels and Cd in blood from Cd-exposed rats [154]. Further, in Cd-exposed rat lungs, the lncRNA ENST00000414355 shows a positive correlation with blood Cd concentration. Interestingly, the silencing of this ENST00000414355 decreased Cd-induced DNA damage through increased expression of essential DNA repair genes such as DDB1, DDB2, OGG1, ERCC1, MSH2, RAD50, XRCC1 and BARD1 [155].

#### 3.4.3 Mercury

Mercury, in its inorganic form (Hg^2+^ ion), has deleterious effects in the kidney, whereas the methylated form of mercury (MeHg) targets the central nervous system (CNS) [156]. In an *in vitro* model of CNS differentiation, Pallocca et al. showed that MeHg chloride treatment of co-cultured neuronal/glial cells during their differentiation promoted differential regulation of several miRNAs (miR-141, miR-196b, miR-302b, miR-367, and miR-372), whose targets were mapped to pathways involved in axonal guidance, learning, and memory [157]. Ding et al. reported that high-level Hg exposure correlates with miR-92a and miR-486 expression, suggesting that these two miRNAs may be potential biomarkers in populations occupationally exposed to Hg [158]. In an independent cohort, a decrease in the levels of miR-575 and miR-4286 in the human cervix was reported as a response to maternal chemical exposure during pregnancy [159].

The lncRNA MALAT1 seems to respond as well to HgCl_2_ and MeHg exposure in the zebrafish embryo. Cao et al. described that MALAT1, among other four candidates, was the only significantly upregulated in the brain and notochord of the fish embryo. They proposed that MALAT1 could represent a potential biomarker for mercury-induced damage [160]. However, more extensive research needs to be done in further cellular and *in vivo* models. Lu et al. studied the correlation between DNA methylation changes due to mercury exposition and three imprinted lncRNAs, H19, MEG3 and PEG3 (paternal expression gene 3), analyzing the sperm and urine from a cohort of 616 men. They found a negative correlation between H19 methylation and mercury levels in urine [161]. As previously mentioned, changes in H19 methylation and imprinting can have severe consequences in fetal growth [70].

### 3.5 Air pollution

#### 3.5.1 Particulate matter

The SPHERE study (Susceptibility to Particle Health Effects, microRNAs, and Exosomes) [162] is focused on evaluating the adverse health effects of air pollution on obese subjects. Rodosthenous et al. found an association between the long-term exposure to ambient particulate matter (PM) of <2.5 μm in diameter (PM2.5) and the increased levels of serum miRNA levels. These included miR-126–3p, miR-19b-3p, miR-93–5p, miR-223–3p, and miR-142–3p with 6 months of exposure, and miR-23a-3p, miR-150–5p, miR-15a-5p, miR-191–5p, and let-7a-5p with 1 year of PM2.5 exposure. Gene targets of these miRNAs were associated with cardiovascular disease as they were important regulators of oxidative stress, inflammation and atherosclerosis [163].Summary of miRNA and LncRNA in air polution can be found on Table 4.

**Table 4:** List of relevant studies in air pollution and ncRNAs from 2014 to present.

	PM size	Target genes	Altered function	Disease	Ref
miRNAs					
**miR-126 ↓**	<2.5	N/A	Oxidative stress, inflammation	Atherosclerosis	[111]
**miR-19b ↓**					
**miR-93 ↓**					
**miR-223 ↓**					
**miR-142 ↓**					
**miR-23a ↓**	<2.5	N/A	Oxidative stress, inflammation	Atherosclerosis	[185]
**miR-150 ↓**					
**miR-15a ↓**					
**miR-191 ↑**					
**let-7a ↑**					
**miR-126 ↓**	<2.5	High-mobility group chromatin proteins	Inflammation	Endothelial dysfunction, atherosclerosis	[112]
**miR-146a ↓**					
**miR-155 ↓**					
**miR-21 ↓**					
**miR-222 ↓**					
**miR-21 ↓**	<10	N/A	Oxidative stress, inflammation	Retinal arteriolar narrowing	[113]
**miR-222 ↓**					
**miR-144 ↓**	<2.5	Zeb1	EMT	Lung cancer	[114]
**miR-4516 ↑**	<2.5	RPL37	Autophagy	Lung cancer	[115]
		UBA52			
**miR-183 ↑**	<2.5	FOXO1	Inflammation	Sperm quality	[116]
**miR-16 ↓**	<2.5	Twist1	EMT	Hepatocellular carcinoma	[117]
**miR-224 ↑**	<2.5	TLR2	Inflammation, airway remodelly	Asthma	[118]
**let-7a ↓**	<2.5	ARG2	Oxidative stress cell injury, apoptosis	Airway epithelial injury	[119]
**miR-32 ↓**	<2.5	Smad1	EMT	Lung cancer	[120]
**miR-194 ↓**	<2.5	DAPK1	Apoptosis	Bronchial epithelial injury	[121]
LncRNAs					
**MALAT1 ↑**	<2.5	Zeb1 miR-204	EMT	Lung cancer	[122]
**LCPAT1 ↑**	<2.5	RCC2	Autophagy	Lung cancer	[123]
			EMT		
**MEG3 ↑**	<2.5	p53	Autophagy, apoptosis proliferation, inflammation	COPD	[[124], [125]]
	CSE	miR-218			
**HOTAIR ↑**	CSE	N/A	EMT	Lung cancer	[126]
**LOC101927514 ↓**	<2.5	STAT3	Inflammation	Bronchial epithelial injury	[127]

**N/A**: not available, **CSE:** cigarette smoke extract.

Changes in miRNA expression have also been explored as biomarkers of air pollution exposure [164,165]. In follow-up studies, Fossati et al. investigated the relationship between miRNAs in peripheral blood leukocytes (PBLs) and PM exposure. The miRNAs miR-1, miR-126, miR-135a, miR-146a, miR-155, miR-21, miR-222, and miR-9 were all associated with PM exposure. Pathway analysis revealed that miR-126, miR-146a, miR-155, miR-21 and miR-222 were strongly associated with changes in the high-mobility group chromatin proteins. The relationships between the expression of these miRNAs and PM exposures were influenced by polymorphisms in the RNA processing genes GEMIN4 and DGCR8 [166]. More recently, Motta et al. found that miRNAs provide a likely regulatory mechanism underlying the BP-related effects of air pollution exposure, showing that changes in miR-101 expression provide an important epigenetic basis for this action [167].

PM has adverse effects on vascular function, as assessed by measuring the impact on BP and flow-mediated vessel dilation [167]. Louwies et al. measured microvascular responses to PM with retinography and explored the role that oxidative stress-mediated induction of miR-21 and miR-222 might have on PM-induced changes in these microvessels. Both miR-21 and miR-222 levels correlated with PM-induced abnormalities in retinal microvessel diameter, suggesting a role for oxidative stress and inflammation in these effects [168]. Although less investigated than the effects on cardiopulmonary function, a recent study documented the association between miRNA expression, air pollution exposure and lung cancer. Pan et al. found that miR-144 levels were decreased in air pollution-related lung cancer, possibly by targeting the oncogene Zeb1 (Zinc finger E-box-binding homeobox 1) [169]. In this condition, miR-4516 regulates autophagy-associated ribosome function genes RPL37 and UBA52 [170], while miR-32 downregulation promotes EMT through the modulation of Smad1 [171]. Particulate matter-induced lung epithelial injury is counteracted by let7 which modulates oxidative stress through arginase 2 [172] and promoted by miR-194 upregulation which control death-associated protein kinase 1 (DAPK1) [173]. Finally, there is evidence that PM is associated with suppression of innate immunity and decreased clearance of viruses. Hou et al. explored the associations of short-term PM2.5, EC, and PM10 with miRNA expression, suggesting that latent viral miRNAs are potential mediators of air pollution-associated health effects [174].

Previous studies found that lncRNAs are also involved in the development and progression of diseases associated with air pollution. Interestingly, the lncRNA MALAT1 is induced with PM *in vitro*. This lncRNA impacts Zeb1 and the EMT process through the downregulation of miR-204 [175]. These two mechanisms contribute to invasion and lung cancer progression. Other lncRNAs were identified in lung cancer induced by PM and cigarette smoke. A lncRNA, named lung cancer progression-association transcript 1 (LCPAT1), increases its expression in *in vitro* studies triggering autophagy and invasion via RCC2 [176]. Similarly, MEG3 upregulation after PM exposure leads to autophagy and apoptosis. Li et al. studied that silencing this specific lncRNA would be beneficial for the treatment of COPD. Disruption of MEG3 expression in human bronchial cells prevented apoptosis and autophagy, potentially through a p53 dependent mechanism [177], while it regulates cigarette smoke extract (CSE)-induced apoptosis and inflammation via miR-218 in 16HBE cells [178]. Moreover, cigarette smoke extracts increase levels of HOTAIR in human bronchial epithelial (HBE) cells [179]. Aberrantly expressed long non‐coding RNAs due to air pollution can also induce congenital defects. He et al., showed the correlation between lncRNA H19 methylation and length and weight at birth. Their data showed that prenatal NO_2_ exposure correlated with increased H19 methylation, whereas PM10 exposure and SO_2_ exposure did so with reduced H19 methylation [180]. Li Z et al., found 554 differentially expressed lncRNAs (216 up‐regulated and 338 down‐regulated) in air pollution‐exposed rat embryos, with potential cellular functions in neurological mechanisms, sensory perception of smell and the G‐protein signaling pathway [181].

#### 3.5.2 Asbestos

Malignant pleural mesothelioma is an aggressive cancer, highly related to exposure to the chemical stressor asbestos, a natural mineral widely used in construction materials. This disease is characterized by a significantly long latency period (40–60 years) after the exposure. The absence of suitable biomarkers for early diagnosis [182] and lack of appropriate treatments for late-stage patients remain challenging. Interestingly, a microRNA has been involved in the progression of mesothelioma. Munson, et al. have described that mesothelioma cells secrete exosomes containing tumor suppressor ncRNAs, like the microRNA miR-16. Reintroducing miR-16 into the mesothelioma cells reduces the tumorogenic capacity and it could be a great therapeutic strategy [183]. MesomiR 1, is a miR-16 based therapy designed as a targomer (miRNA therapeutic delivery with nanoparticles) that is under Phase I clinical trial (NCT02369198) [184,185]. Some efforts have also been done to test changes in lncRNAs in this model, trying to find new early biomarkers for this model. Several lncRNAs, AK130275, AK129685, EF177379, BX648695, NR_003584, and AF268386 were upregulated in mesothelioma tissue compared to healthy pleura. This lncRNA panel is highly sensitive and has great potential as a biomarker for mesothelioma [186]. Another lncRNA involved in mesothelioma is GAS5, with a reported low expression in mesothelioma cells. Growth arrest was able to induce GAS5 expression and accumulation in the nuclei, leading to quiescent tumor cells [187].

### 3.6 Pesticides

The use of pesticides is intended for the prevention or mitigation of different pests like insects, rodents, or weeds. However, its exposure can be deleterious for humans [188]. Organophosphates, which are a group of insecticides that inhibit acetylcholinesterase, promote cognitive dysfunction in the learning and memory process. miR-132 and miR-212 have been proposed to mediate the disruption of neurotrophin-mediated cognitive processes after chlorpyrifos exposure [189]. Urinary miRNAs have been suggested as potential biomarkers for human exposure [190]. The organophosphate dichlorvos induces aberrant expression of miRNAs, inducing both neurotoxicity and non-neuronal toxicity [191]. Conazole-dependent formation of liver cancer is mediated by miRNA dysregulation [192]. Paraquat produces toxicity in the lung through superoxide anion formation and eventually hydroxyl radicals leading to lipid peroxidation [193]. In human neural progenitor cells, paraquat treatment alters microRNA expression modulating neural proliferation, differentiation, cell cycle and apoptosis [194].

Few studies have been performed to study the impact of pesticide exposure on lncRNAs. Some preliminary lncRNA expression profiles have been described with the insect repellent N,N-diethyl-m-toluamide (DEET) and the insecticide fluocyanobenpyrazole (fipronil). Mitchell et al. showed that 20 lncRNA are commonly dysregulated in response to these two insecticides independently or when used in combination in primary human hepatocytes. They observed than 18 of these lncRNAs were downregulated, and only 2 of them were increased [195]. The most upregulated lncRNA from this list was CYP2B7, a pseudogene from the CYP450 family.

Paraquat is a widely used herbicide. The molecular mechanism of paraquat depends on redox cycling and superoxide anion formation. Paraquat accumulates in the lungs leading to pulmonary fibrosis [193]. In a mouse model of paraquat-induced pulmonary fibrosis, two upregulated lncRNAs, uc.77 and 2700086A05Rik, were involved in modulation of EMT. These lncRNAs target Zeb2 and HOX3, critical genes in the initiation of EMT [196]. In the brain, Paraquat poisoning acts as a neurotoxin, and it has been associated with higher risk of Parkinson's disease [197,198]. In this context, paraquat also alters lncRNAs in the mouse susbtantia nigra through interaction with the transcription factor Nrf2 [199]. However, the functional role of this lncRNA needs further exploration.

## 4 Future perspectives and conclusions

A rapidly growing number of ncRNAs has been identified in the last three decades due to developments in genomics and bioinformatics. However, besides efforts have been made to model the complexity of the networks and regulatory mechanisms of ncRNAs involved in environmental pollution gene regulation, in the overwhelming majority of cases definitive or causative roles for these exposure-disease associations have not been established. Notwithstanding, they have provided the opportunity to explore selected ncRNAs as potential biomarkers of toxic exposure, as well as potential therapeutic targets. Based on new oligonucleotide therapies under U.S. Food and Drug Administration (FDA) approval and several microRNAs-based therapies clinical trials, we have attempted to summarize the most favorable cases of microRNAs as potential therapeutic agents. Among them, mesomir is a promising drug for malignant pleural mesothelioma, a lethal disease with yet a lack of effective treatment. The mesomir clinical trial is awaiting to start Phase I, after successful studies in *in vivo* experimental models. Still the concern with mesothelioma is the latency state after asbestos exposure, hence the need of identifying a good combination of ncRNA biomarkers.

In the last years, our understanding of microRNA biology and their influence on regulation of gene expression has grown and brought new actors, in particular lncRNAs. These molecules may adopt different roles depending on their topology, interactions and cell types. LncRNA structure transforms junk-RNA molecules into versatile modifiers of gene expression and molecular function. They are promiscuous molecules that can partner either with nucleic acids or proteins. LncRNA expression is low within the cells and their own expression is tightly regulated. These molecules have demonstrated essential roles in development and gene-dosage and are highly conserved across mammals. Xist lncRNA silences one of the X-chromosomes in females inactivating gene expression. Mutation in this lncRNA can lead to incomplete X-chromosome inactivation favoring X-linked diseases such as Duchenne muscular dystrophy or hemophilia A [200].

Fluctuations of lncRNAs due to environmental toxins in utero or germ line can lead to dramatic developmental and growth defects in the fetus. Different agents such as oxidative stress, BPA and mercury affect DNA methylation of the imprinted lncRNA H19. H19 has been linked to pathologies like diabetes. However, H19 expression changes in germline or in utero after toxic exposure can cause severe damage in fetus development [70].

We herein featured several lncRNAs that are commonly upregulated with specific stressors. Many of them have been recently described as high-confidence lncRNAs in the Pan-Cancer Analysis of Whole Genomes (PCAWG) study [201]. Among them is the case of MALAT1. MALAT1 responds to changes in the redox balance, such as treatment with hydrogen peroxide or hypoxia. Not surprisingly, this lncRNA is also affected after UV, air pollutants mercury, cadmium or arsenic [32,45,139,154]. MALAT1 is regulated by Nrf2/Keap1 antioxidant response. Independently of the mechanism behind MALAT1 expression, increased expression seems to be deleterious, promoting cancer progression and metastasis though activation of EMT pathways [139,175]. Although MALAT1 could be a good prognostic factor for malignant transformation, it unlikely constitutes a good specific biomarker to identify different poisoning agents. The combination of a lncRNA panel might prove more powerful, as exemplified in mesothelioma [186] or Parkinson's disease in early stages [199].

A long road lies ahead to clarify the benefits of lncRNA as potential therapeutic targets. First, the majority of the studies and lncRNA screening here described have been done *in vitro*, with some exceptions *in vivo* and few patient studies. Second, even though we described highly conserved lncRNA among mammals due to the different number of mechanisms in several cell lines, it is still difficult to predict whether external overexpression or inhibition would be beneficial in pathological progression. However, in-depth knowledge of the downstream mechanisms of these lncRNAs may help to design new therapeutic strategies to prevent and control deleterious consequences of toxic environmental stressors. Therefore, experimental and population-based epidemiological studies are warranted to clarify lncRNAs role in the interaction of genetic and environmental factors underpinning the development of many common diseases.

## Declaration of competing interest

The authors declare that they have no known competing interests or personal relationships that could have appeared to influence the work reported in this paper.
